# Comparative Mitogenomic Analysis of Two Cuckoo Bees (Apoidea: Anthophila: Megachilidae) with Phylogenetic Implications

**DOI:** 10.3390/insects12010029

**Published:** 2021-01-05

**Authors:** Huanhuan Lu, Bo He, Youjin Hao, Zeyang Zhou, Chengyong Su, Dunyuan Huang

**Affiliations:** 1 Chongqing Key Laboratory of Vector Insects, College of Life Sciences, Chongqing Normal University, Chongqing 401331, China; 2019110513046@stu.cqnu.edu.cn (H.L.); 20130100@cqnu.edu.cn (Y.H.); zyzhou@cqnu.edu.cn (Z.Z.); 2 College of Life Sciences, Anhui Normal University, Wuhu 241000, China; hebo90@ahnu.edu.cn

**Keywords:** Megachilidae, mitochondrial genome, genome structure, phylogenetic analysis, gene rearrangement

## Abstract

**Simple Summary:**

Megachilidae plays an important role in natural and agricultural ecosystems. There is uncertainty in the phylogenetic relationship among tribes of Megachilidae. Due to the lack of basic analysis of the mitogenomic structure of the cuckoo bees (cleptoparasitic bees) of the Megachilidae, the risk of insect cleptoparasitism in the Megachilidae is not fully understood. To further provide a new perspective on the phylogenetic relationship of Megachilidae and enrich the basic theory of cleptoparasitic controls, two mitogenomes of cuckoo bees (*Coelioxys fenestrata* and *Euaspis polynesia*) were sequenced and analyzed. Different mitogenomic structures and base compositions were found between two cuckoo bees based on comparative analyses of general characteristics of the mitochondrial, noncoding region and gene rearrangement pattern. In addition, the phylogenetic results strongly supported that the tribe-level relationship of Megachilidae was Osmiini + (Anthidiini + Megachilini). Our findings clarified the phylogenetic position among tribes from the mitogenome level so as to provide a further basis to study the evolution of Megachilidae.

**Abstract:**

Bees (Hymenoptera, Apoidea and Anthophila) are distributed worldwide and considered the primary pollinators of angiosperm. Megachilidae is one of the largest families of Anthophila. In this study, two complete mitogenomes of cuckoo bees in Megachilidae, namely *Coelioxys fenestrata* and *Euaspis polynesia*, were amplified and sequenced, with a length of 17,004 bp (*C. fenestrata*) and 17,682 bp (*E. polynesia*). The obtained results show that 37 mitogenomic genes and one putative control region were conserved within Hymenoptera. Truncated stop codon T was found in the *cox3* gene of *E. polynesia*. The secondary structure of small (*rrnS*) and large (*rrnL*) rRNA subunits contained three domains (28 helices) and five domains (44 helices) conserved within Hymenoptera, respectively. Compared with ancestral gene order, gene rearrangement events included local inversion and gene shuffling. In order to reveal the phylogenetic position of cuckoo bees, we performed phylogenetic analysis. The results supported that all families of Anthophila were monophyletic, the tribe-level relationship of Megachilidae was Osmiini + (Anthidiini + Megachilini) and *Coelioxys fenestrata* was clustered to the Megachile genus, which was more closely related to *Megachile sculpturalis* and *Megachile strupigera* than *Euaspis polynesia*.

## 1. Introduction

Megachilidae, one of the largest families in Anthophila, comprises about 4000 species and is distributed almost all over the world [1,2,3]. It is characterized by most female bees using their upper jaws to cut leaves as nesting materials [4,5]. The extant Megachilidae, most widely accepted, is divided into two subfamilies and seven tribes [1]. Megachilidae can be further classified as pollinating bees with abdominal brushes and cuckoo bees (cleptoparasitic bees) without abdominal brushes [1,6]. Pollinating bees play an important role in nature. For example, *Megachile rotundata* (Fabricius, 1793) has been promoted in all parts of the world as an *alfalfa* (Medicago sativa L.) pollinator [7], *Osmia lignaria* (Say, 1837) has a better pollination effect than *Apis mellifera* (Linnaeus, 1758) (Hymenoptera: Apidae) [8] and Megachilid bees have been found all over the world except Antarctica [6]. Therefore, as pollinators, they play an irreplaceable role in maintaining the ecosystem and increasing the yield of many crops [5,6,9].

Megachilidae contains many cuckoo bees that attack pollinators of other tribe-genera of the same family [1,6]. Cleptoparasitism means that cleptoparasitic bees lay their eggs on pollen clusters or egg chambers made by pollinating bees, and their larvae grow on the food provided by the host. Compared with *C. fenestrata*, the female bees of *E. polynesia* sneak into the closed nest of the host for cleptoparasitic activities, which causes the loss of a large number of pollinators [5]. At the same time, they are easy to catch and have a wide range of hosts. Cleptoparasitism of insects of the same family is a rare phenomenon. For instance, the *Euaspis* genus (Gerstacker, 1858) cleptoparasitizes the genera *Lithurgus* (Berthold, 1827) and *Megachile* (Latreille, 1802) [10,11], and the *Coelioxys* genus (Latreille, 1809) is cleptoparasitic in all genera of the tribe Megachilini [12,13]. In addition, some studies suggested that all tribes of the Megachilinae subfamily are monophyletic, and the tribe-level phylogenetic relationships were proposed as Anthidiini + Megachilini + Osmiini [14,15]. However, other studies suggested that some species of Megachilini originated from Osmiini, Osmiini are considered paraphyletic [1,16,17].

Mitochondrion is a semiautonomous organelle, in which oxidation is performed and energy is released for eukaryotes. A typical insect mitochondrial genome (mitogenome) contains 13 protein-coding genes (PCGs) encoding protein subunits involved in oxidative phosphorylation, 22 tRNAs (*trnS* and *trnL* have two isomers) and two rRNAs (*rrnL* and *rrnS*) [18]. In addition, the insect mitogenome has a control region (CR) [19] that regulates replication and transcription. Because of distinctive properties such as maternal inheritance, strict orthologous genes and a low rate of recombination [20,21], insect mitogenomes have been extensively applied for intraordinal phylogen [22,23,24], phylogeography [25] and molecular evolution [26,27] as a molecular marker.

Currently, only complete mitogenomes of three pollinating bees of Megachilidae have been released in the National Center for Biotechnology Information (NCBI) [28,29,30]. In this study, we sequence the mitogenomes of the two cuckoo bees and analyze the differences of mitogenomes between the two species from their general characteristics, genome structure, special structure of the noncoding region and gene rearrangement pattern. At the same time, we also perform phylogenetic analysis in order to clarify the interspecific relationship of Megachilidae and the phylogeny of Anthophila for short- and long-tongued bees. Overall, our results provide a basis for further phylogenetic analysis of cuckoo bees in the Megachilidae family.

## 2. Materials and Methods

### 2.1. Sampling and DNA Extraction

Adult stages of *C. fenestrate* and *E. polynesia* were collected by the sweeping net method in the Jinggang Mountains, Jiangxi Province, China, in October, 2019. The latitude and longitude of the collection sites are 26°28′16.5′′ N and 114°12′33.8′′ E, identified by Dr. Ze-qing Niu (Institute of Zoology, Chinese Academy of Sciences) based on morphological characteristics. The voucher specimens (No. *C. fenestrate*—2019-2T-1 and No. *E. polynesia*—2019-2E-1) were stored at −20 °C (College of Life Sciences, Chongqing Normal University). Total genomic DNA of one adult per species was extracted with the Tissue DNA Kit (Omega Biotek, Norcross, GA, USA) following the manufacturer’s instructions.

### 2.2. Sequencing and Assembly

The mitochondrial DNA was fragmented to an average size of 450 bp using the Covaris M220 system (Covaris, Woburn, MA, USA) and used for the library preparation. The library was constructed using the Illumina TruSeq™ Nano DNA Sample Prep Kit (Illumina, San Diego, CA, USA) and sequenced on the platform of Illumina Hiseq 4000 (Illumina, San Diego, CA, USA). Before assembly, raw reads were filtered, and quality was assessed using Fast-QC (http://www.bioinformatics.babraham.ac.uk/projects/fastqc). High-quality clean reads were used for the subsequent analysis based on Q20 (>95%) and Q30 (>90%). The complete nucleic acid sequence was assembled by MITObim v1.7 [31] based on the reference sequence of *Osmia excavata* (Alfken, 1903) [30] (GenBank accession number: KX494106).

### 2.3. Bioinformatic Analysis

The tRNAs and their secondary structures were identified using the MITOS web server [32], ARWEN [33], tRNAscan-SE Online Search Server [34] and by manual proofreading. Boundaries of PCGs were determined by the positions of tRNAs. PCGs were predicted using the Open Reading Frames (ORFs) finder implemented in Unipro UGENE v34 [35] and confirmed by the MITOS web server. Similarly, positions of rRNAs and the CR were identified based on the boundaries of tRNAs and homology comparison [28,29]. The secondary structure of rRNAs (*rrnL* and *rrnS*) was deduced from the known models of other Hymenoptera insects [36,37,38,39]. Helix numbering was determined to be in accordance with the regulations of the comparative RNA web (CRW) [40]. Moreover, the secondary structure was folded through RNA Structure v5.6 [41] and identified by manual proofreading. Finally, the comparable gene identical map was visualized by the BLAST Ring Image Generator (BRIG) v0.95 [42].

Base composition, AT or GC skews, codon usage and relative synonymous codon usage (RSCU) were analyzed by PhyloSuite v1.2.2 [43]. DnaSP (v6.12.03) was applied to estimate the nucleotide diversity (Pi) between the mitogenomes of *C. fenestrata*/*M. sculpturalis* (Smith, 1853) and *E. polynesia*/*M. strupigera* (Cockerell, 1922) [44]. The tandem repeats in the intergenic spacers were predicted using the Tandem Repeats Finder program (http://tandem.bu.edu/trf/trf.basic.submit.html). The CR was searched through the MISA online web server (MIcroSAtellite identification tool) [45].

### 2.4. Phylogeny Analysis

To explore their phylogenetic relationships, 27 mitogenomes of Anthophila and two outgroup species, *Abispa ephippium* (Fabricius, 1775) (Hymenoptera: Vespidae) and *Philanthus triangulum* (Fabricius, 1775) (Hymenoptera: Crabronidae), were used (Table S1). Multiple sequence alignment of 13 PCGs, 22 tRNAs and two rRNAs was performed by Mafft v7.310 in PhyloSuite (alignment strategy: L-INS-i). Individual alignments were then concatenated using PhyloSuite, and the poorly aligned and high divergence regions were removed by Gblocks 0.91b in PhyloSuite. In addition, the potential index of substitution saturation (*Iss*) of each nucleic acid sequence was calculated by DAMBE v7.2.1 [46]. To test the influence of the 3rd codon and gene combination types on the subsequent phylogenetic analysis, four datasets were constructed: (1) the 1st and 2nd codon positions of 13 PCGs and 22 tRNAs (PCG_12_+T); (2) all three codon positions of PCGs and tRNAs (PCG_123_+T); (3) the PCG_12_+T and two rRNAs (PCG_12_+T+R); (4) total gene sequences (PCG_123_+T+R). Partition-Finder (v2.1.1) was used to infer the best evolutionary model [47]. Finally, the evolutionary processes of gene arrangement in two cuckoo bees were estimated by the Common-interval Rearrangement Explorer (CREx) [48].

The BI and ML trees were constructed by MrBayes v3.2.6 and IQ-TREE v1.6.8 within PhyloSuite, respectively. The ML analysis was conducted under the parameters of an ultrafast bootstrap with 1000 replicates. The BI analysis was conducted with four Markov Chain Monte Carlo (MCMC) chains of 1 million generations twice, which was sampled every one thousand steps and discarded the first 25% of the generations as burn-in. When the average standard deviation of the split frequency was less than 0.01, the potential scaling reduction factor (PSRF) was close to 1, and when the estimated sample size (ESS) was greater than 200, the MCMC analysis was stopped. The ESS value was viewed through Tracer v1.7.1 [49]. Phylogenetic trees were visualized and edited by iTOL [50].

## 3. Results and Discussion

### 3.1. General Features of the Mitogenome of C. fenestrata and E. polynesia

**Figure 1:**
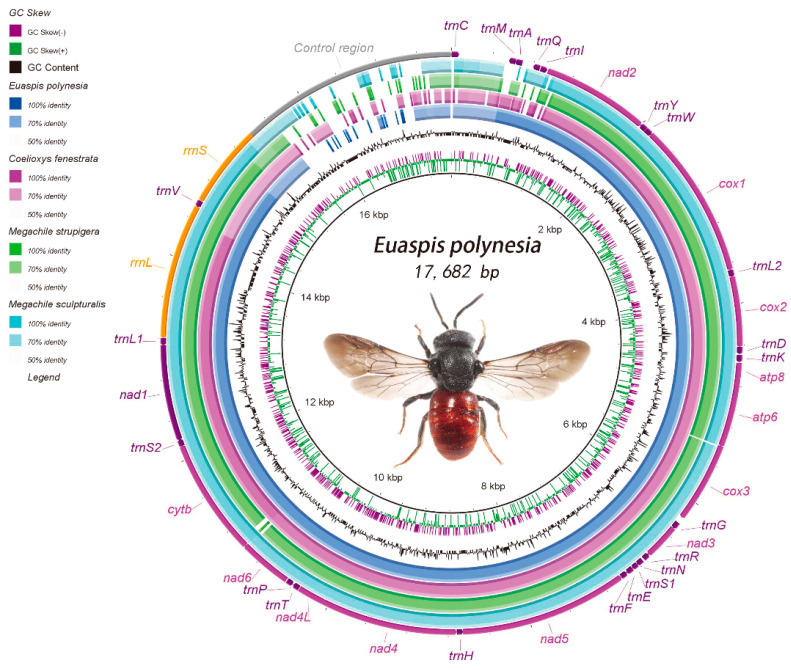
Circular map of the mitogenome of Megachilidae. The different colors represent the nucleotide identity using the mitogenome of *O. excavata* as a reference (Acc. No.: KX494106) [30].

The complete mitogenome of *C. fenestrata* (GeneBank accession number: MT898425) and *E. polynesia* (MT909816) is 17,704 bp and 17,682 bp, respectively (Table S1). Each contains 37 typical mitogenomic genes [29,51]. Most are concentrated at the J strand (9 PCGs, 13 tRNAs of *C. fenestrata* and 12 tRNAs of *E. polynesia*). Other genes (4 PCGs, 2 rRNAs, 9 tRNAs of *C. fenestrata* and 10 tRNAs of *E. polynesia*) are concentrated at the N strand (Figure 1, Table S2). For *C. fenestrata*, the total nucleic acid sequence of all PCGs is 11,151 bp with 79.6% AT content, and the length/AT content of tRNAs and rRNAs is 1423 bp/85.7% and 2064 bp/84.9%, respectively (Table S3). Except for the control region, 18 intergenic regions were found in the mitogenome of *C. fenestrata* (390 bp totally) and *E. polynesia* (1032 bp totally). Nine and ten overlapping regions were found in the mitogenome of *C. fenestrata* (39 bp totally) and *E. polynesia* (58 bp totally), respectively (Table S2).

Compared with three publicly released mitogenomes of Megachilidae (*O. excavate*, *M. sculpturalis* and *M. strupigera*), *C. fenestrata* has the lowest AT content (82.9%), and *E. polynesia* has the highest AT content (85.9%) (Table S1). From the bias skew, the AT and GC skews in both mitogenomes are similar to those of other Megachilidae species: more A/C than T/G in the J strand [52], positive AT skew and negative GC skew (Table S1). The mitogenome of Megachilidae was visualized so that a circular map was generated (Figure 1). The result showed that the locations of some tRNAs (e.g., *trnE*, *trnF*, *trnK*, *trnL2*, *trnP*) are highly conservative. For PCGs, genes *cox1*, *cox2* and *cox3* are more conservative than genes *nad2*, *nad4L*, *cytb* and *nad6* (Figure 1).

### 3.2. Genome Structure

#### 3.2.1. Protein-Coding Genes

In both newly sequenced mitogenomes, the usage patterns of the start codon and the stop codon are similar (Table S2). For instance, the most frequently used start codon by PCGs is ATN. Although most PCGs use TAA or TAG as the stop codon, a truncated stop codon T was found in the *cox3* gene of *E. polynesia*. Truncated stop codons are commonly observed in many mitogenomes of Hymenoptera insects and are expected to be completed via the post-transcriptional polyadenylation process [53].

**Figure 2:**
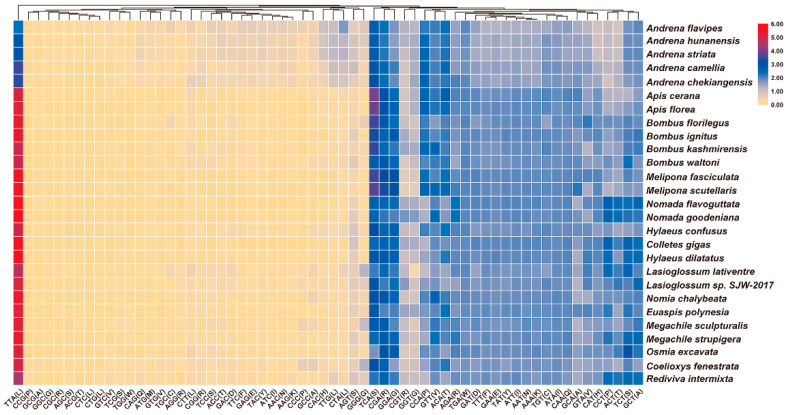
The relative synonymous codon usage (RSCU) of protein-coding genes (PCGs) in the mitogenomes of Anthophila. The *x*-axis and *y*-axis represent the codon type and species name, respectively. The legend in the upper-right corner represents the usage frequency of synonymous codons.

The result of RSCU analyses showed that the frequency of A/T is higher than that of G/C in the third codon position (Figure 2). For instance, the third codon position among the six most commonly used codons (TTA, TCA, CGA, ACA, GTT and TCT) in the mitogenome of *C. fenestrata* is A or T. On the contrary, codons rich in C or G are rarely used in the mitogenome of *C. fenestrata*, such as CCG, GCG, GGC and CGC. This phenomenon is more obvious in the mitogenome of *E. polynesia* because codon CGC and GCG are not used at all (Table S4).

#### 3.2.2. Transfer RNA Genes

**Figure 3:**
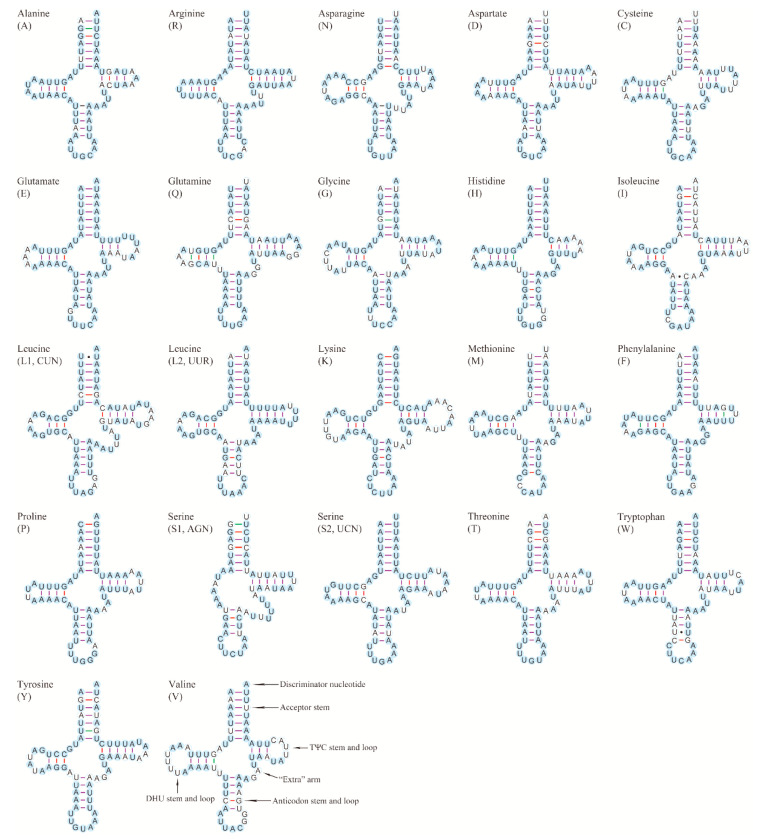
Secondary structures of tRNAs in the mitogenome of *C. fenestrata*. The filled circle represents the nucleotide conserved. The bonds of A-U, G-U, G-C and mismatches are marked with a purple line, green line, red line and solid dots, respectively.

Twenty-two typical tRNAs were identified in both mitogenomes (Figure 3). Most of them could be folded into a typical clover-leaf secondary structure except for *trnS1*. This phenomenon often occurs in the mitogenomes of Hymenopteran insects [36,37,54]. The secondary structure of tRNAs consists of “four arms” and “four loops.” Among them, the amino-acid arm (14 bp) and anticodon loop (7 bp) are highly conserved, which is very common for metazoans [38,39]. The extra loop determines the molecular weight of tRNAs. Moreover, the secondary structures of tRNAs contain some unconventional base pairs such as G-U specific matches and A-C or G-U mismatches, which were also found in the mitogenomes of other Hymenoptera insects [36,37,38,39]. Finally, these unconventional structures will be corrected in the subsequent editing stage [55] or represent unusual pairings [40].

#### 3.2.3. Ribosomal RNA Genes

Compared with the mitogenome of *E. polynesia*, the sequence alignments of *rrnL* of *C. fenestrata* spans 1342 sites including 1026 conserved sites (76.45%) and 361 variable sites (23.55%) (Figure S1). Furthermore, the sequence alignments of *rrnS* spans 805 sites including 615 conserved sites (76.40%) and 190 variable sites (23.60%) (Figure S2). Conserved nucleotides were dispersed in each rRNA sequence.

The secondary structure of *rrnS* and *rrnL* contains three domains (28 helices) and five domains (44 helices), respectively. Among the five domains of *rrnL*, Domain III does not exist, which is a typical feature of arthropods [40]. The three domains of *rrnS* have always been controversial. For example, the nonconservation of helix H47 leads to the variousness of the secondary structure of Domain I in insects [56]. Although helix H673 forms a relatively conservative structure in Hymenoptera [36,37,38,39], it shows diverse secondary structure models in other insect species [57,58].

#### 3.2.4. Noncoding Regions

**Figure 4:**
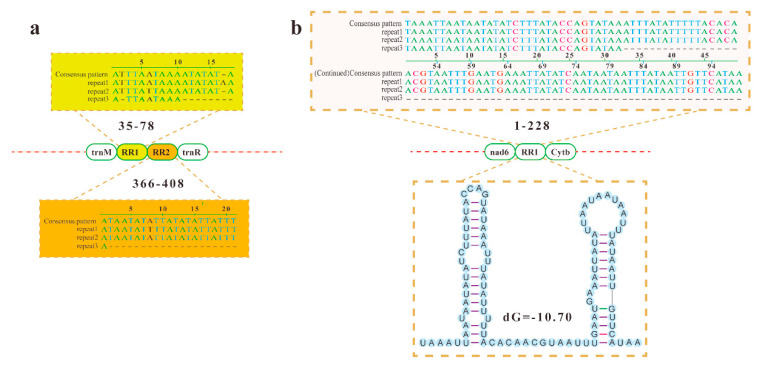
Repetitive sequences and secondary structure of intergenic spacers (IGSs) of the mitogenome of *E. polynesia* (**a**) and *C. fenestrata* (**b**). The numbers above/below the boxes denote the positions of the RRs within the IGSs.

The noncoding regions of the mitogenome consist of the intergenic spacers (IGSs) and the control region (CR). The IGSs of the mitogenome of *E. polynesia* (469 bp) are located between genes *trnM* and *trnR* with two repeat regions (RRs) (Figure 4a). RR1 consists of three tandem repeats (44 bp totally) with insertions and deletions. RR2 is also composed of three tandem repeats (43 bp totally) with base mutations (Figure 4a). The IGSs of the mitogenome of *C. fenestrata* are located between genes *nad6* and *cytb* and also contain a region (228 bp) with three tandem repeats (Figure 4b). Except for nucleotide deletions, no mutations and insertions were found. Further analysis revealed that the IGSs of the mitogenome of *C. fenestrata* form a secondary structure (Figure 4b). Similar repeat sequences and complementary secondary structures were also found in IGSs of the mitogenomes of other species [59,60]. The secondary structure formed by repetitive sequences is usually related to the starting of the replication of the mitogenome [61,62].

The AT content in the CR is 90% for *C. fenestrata* (2015 bp) and 91.4% for *E. polynesia*, (2128 bp), respectively, which was higher than in other regions. In the CR of the mitogenome of *C. fenestrata*, AT and GC skews are −0.020 and −0.420, respectively. Furthermore, for *E. polynesia*, A/G are more abundant than T/C (AT skew = −0.082, GC skew = 0.402). Further analysis showed that there were many repetitive sequences similar to microsatellites in the CR (Table S5). Microsatellite-like sequences among individuals in different geographical locations were proposed as a new marker to study the phylogenetic geography of Hymenoptera [63].

#### 3.2.5. Gene Rearrangement

**Figure 5:**
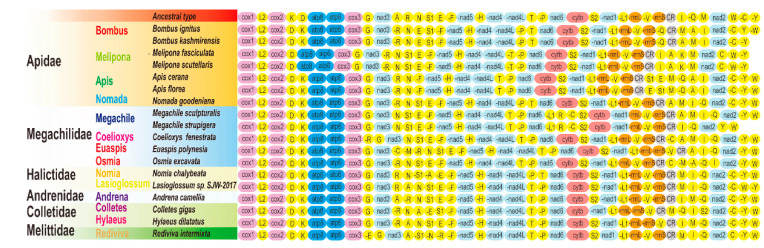
Gene arrangement of the mitogenomes. “-” indicates that the gene is encoded by the N strand; otherwise, they are encoded by the J strand. The three columns to the left of the gene rearrangement are the family, genus, and species of bees.

Gene rearrangement can be divided into remote inversion, local inversion, translocation and gene shuffling [52]. Local inversion accounts for a large proportion of gene order patterns in the mitogenomes of Hymenoptera [64]. In the mitogenomes of Megachilidae, gene shuffling *(trnW-trnC-trnY*, *nad6-cytb-trnS2*, *trnG-nad3-trnA-trnR*, *trnV-rrnS-trnI* and *trnK/trnD*) and a local inversion of *trnR* were found (Figure 5). Similarity analysis showed that the gene order of the mitogenomes of Megachilidae was significantly different (Table S6). Compared with the ancestral gene order of insect mitogenomes, the transformation process from the ancestor to *E. polynesia* and *C. fenestrata* has undergone rearrangement and transposition events (Figure 5 and Figure S3). These patterns were also found in the other 18 species. Gene rearrangements occur in both tRNAs and rRNAs except for PCGs. Rearrangements are concentrated in some regions of tRNAs, including *trnI-trnQ-trnM*, *trnW-trnC-trnY*, and *trnA-trnR-trnN-trnS1-trnE-trnF*, which is also considered a common rearrangement region of other Hymenoptera [65,66]. In addition, another gene rearrangement cluster, *rrnL-rrnS-trnV*, was observed in *E. polynesia*, *Colletes gigas* (Cockerell, 1918) (Hymenoptera: Colletidae) and *Hylaeus dilatatus* (Kirby, 1802) (Hymenoptera: Colletidae) (Figure 5).

### 3.3. Nucleotide Diversity

**Figure 6:**
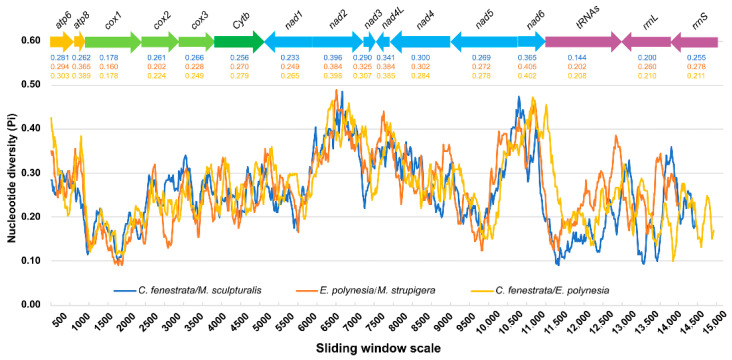
Nucleotide diversity of the mitogenome of *E. polynesia* and *C. fenestrata*. Sequence alignments of 13 PCGs, 2 rRNAs and 22 tRNAs were analyzed by sliding window (window size = 200 bp, step size = 20 bp). The polyline represents the value of nucleotide diversity. The arrow represents the direction of gene coding, with the gene name above it and the average nucleotide diversity value of the gene below.

The results showed that the Pi value ranges from 0.100 to 0.473 in two mitogenomes of *C. fenestrata* (Smith, 1873) and *E. polynesia* (Vachal, 1903) (Figure 6, Table S7). The diversity of genes *nad6* (Pi = 0.402), *nad2* (Pi = 0.398), *atp8* (Pi = 0.389) and *nad4L* (Pi = 0.385) was higher, whereas that of *cox1* (Pi = 0.178), *rrnL* (Pi = 0.210) and *rrnS* (Pi = 0.211) was lower (Table S7: Pi of Gene). To validate the reliability and versatility, further analysis was conducted for genes in the mitogenomes of *C. fenestrata*/*M. strupigera* (Cockerell, 1922) and *E. polynesia*/*M. sculpturalis* (Smith, 1853). Similar results were found in the pairwise comparison of *C. fenestrate* (Smith, 1873) and *E. polynesia* (Vachal, 1903) (Figure 6). All results support that *cox1* is the least variable and can be a potential marker for species identification [67,68]. However, hypervariable genes (*nad6*, *nad2*, *atp8* and *nad4L*) are suitable for studying the phylogenetic relationship of species-level Megachilidae [69].

### 3.4. Phylogenetic Analysis

#### 3.4.1. Substitution Saturation Tests

Substitution saturation of nucleic acid sequences was performed (Table S8). For datasets not filtered by Gblocks, the substitution saturation index (*Iss*) for both the first codon and all codons of PCGs is less than the index of *Iss.cSym* but larger than *Iss.cAsym*. This result suggested that unfiltered datasets may produce some noises for subsequent phylogenetic analysis. It was worth noting that the index *Iss* of the third codon of all PCGs, rRNAs and tRNAs are larger than the index of *Iss.cSym* and *Iss.cAsym*, suggesting that they cannot provide useful information for the phylogenetic analysis. Furthermore, the filtered datasets, except for the third codon and rRNAs, were helpful to explore phylogenetic relationships.

#### 3.4.2. Topology Consistency Analysis

**Figure 7:**
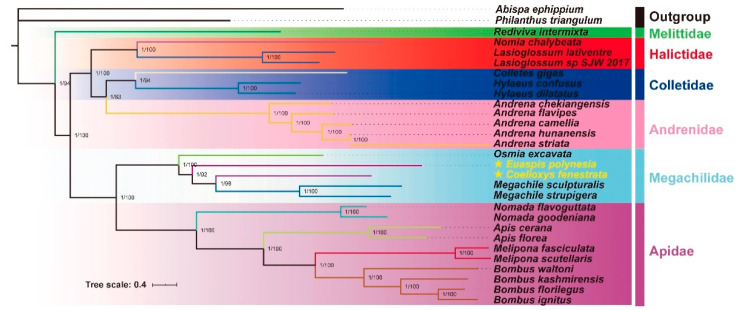
The consensus phylogenetic tree of 27 Anthophila species. The support values of the corresponding nodes are represented by Arabic numerals, the left side of “/” is bootstraps of BI, and the right side of “/” is the posterior probability of ML.

To test the influence of different genes on the phylogenetic analysis, four datasets (PCG_12_+T, PCG_12_+T+R, PCG_123_+T and PCG_123_+T+R) were used (Figure S4). The ML and BI trees are shown in Figure S5. Except for dataset PCG_12_+T+R, the other three datasets produced a consistent topology in both ML and BI analysis (Figure 7). In this study, when the third codons of PCGs were included, the rRNAs data would reduce the node support (e.g., PCG_123_+T+R and PCG_123_+T) and had negative effects on both topology and node support when the datasets excluded the third codons (e.g., PCG_12_+T+R and PCG_12_+T). Interestingly, the negative effect of rRNA genes can be reversed by including the third codon of PCGs. Previous studies also showed that nucleotide sequences of tRNAs could ameliorate node support and the stability of topology [70,71]. In addition, some studies proposed that excluding the third codon can produce a more consistent topology [72], but our results supported that the third codon positions of all PCGs were useful for inferring phylogenetic relationships among taxa that diverged relatively recently [73,74,75].

#### 3.4.3. Phylogenetic Relationship

The phylogenetic analysis based on mitogenomes of 27 species showed that Melittidae, Halictidae, Colletidae, Andrenidae, Megachilidae and Apidae families were a monophyletic group with high support (Figure 7). Species in each family were clustered into one group, except for the Melittidae family, in which only one mitogenome was included. Melittidae was identified as a sister of other families. In related studies, the above conclusion supported the discussion that the Melittidae were the sister group of other bee families of Zheng et al. [30], and supported the family-level hierarchical phylogeny of Anthophila of He et al. [52] and Aydemir et al. [76]. The phylogenetic relationship of Anthophila was (Apidae + Megachilidae) (long-tongued bees) and (Andrenidae + (Halictidae + Colletidae)) (short-tongued bees). The analysis results do not support that the Melittidae belong to the short-tongued bees’ group [18,30] or the three-way split evolution among Melittidae, Andrenidae and the remaining families [77]. Our results were strongly supported by large-scale morphological data [2], multigene tandem sequence [78,79,80], transcriptome [81] and genome [82,83].

In this study, two cuckoo bees were used in the phylogenetic analysis and the results indicated that the *O. excavate* (tribe Osmiini) was a sister group of other species of Megachilidae (Figure 7). Previous phylogenetic analysis based on mitogenomes also revealed that tribe Osmiini was the sister group of other species of Megachilidae [30,52]. *Coelioxys fenestrata*, *M. sculpturalis* and *M. strupigera* formed a group (tribe Megachilini), which was close to *E. polynesia* (tribe Anthidiini). The tribe-level phylogenetic relationship of Megachilidae was Osmiini + (Anthidiini + Megachilini). Although this study is inconsistent with some previous studies [14,15,16,17], the monophyly of each tribe is highly supported in this study. The phylogenetic status of Megachilidae is established with higher supports on each node, which indicates that its phylogenetic relationship is reliable. For the origin of cleptoparasitism within Megachilidae, Michener proposed there were 10 origins [1], whereas Litman et al. thought it should be five or six origins [84,85] because the monophylic status of *Coelioxys* and *Radoszkowskiana* is controversial. Additionally, this cleptoparasitic behavior evolved from a closed nest to an open nest. Therefore, it was considered a unidirectional evolution [84]. In this study, genera *Coelioxys* and *Euaspis*, which contain *C. fenestrata* and *E. polynesia,* respectively, were regarded as independent origins, according to Litman’s opinions about the evolution of cleptoparasitism [84]. Only the mitogenomes of five species are included in this study, and more species will be needed to analyze the phylogenetic relationships of Megachilidae.

## 4. Conclusions

The complete mitogenome sequences of *C. fenestrata* and *E. polynesia* are provided. Comparative genomics and phylogenetic analysis are carried out among the mitogenomes of two cuckoo bees. The results show that a truncated stop codon T is found in *cox3* of *E. polynesia*, which is expected to be completed via the post-transcriptional polyadenylation process. The secondary structures of tRNAs contain unconventional base pairs, which will be corrected in the subsequent editing stage. Gene rearrangement events include local inversion and gene shuffling. The phylogenetic results support that *C. fenestrata* was more closely related to *M. sculpturalis* and *M. strupigera* than *E. polynesia*. The tribe-level relationship of Megachilidae is Osmiini + (Anthidiini + Megachilini). The phylogenetic status of the cleptoparasitism of Megachilidae was more clearly understood. In addition, regarding the sister relationship between Melittidae and other bee families, the other families were divided into two groups: (Apidae + Megachilidae) (long-tongued bees) and (Andrenidae + (Halictidae + Colletidae)) (short-tongued bees). In future studies, more mitogenomes of other species are needed to further explore the phylogenetic relationship of Megachilidae.

## Data Availability

All mitogenomic sequences in this study are available in the GenBank database (https://www.ncbi.nlm.nih.gov/nuccore), and accession numbers of mitogenomes are available in the Supplementary Material (Table S1).

## Supplementary Materials

The following are available online at https://www.mdpi.com/2075-4450/12/1/29/s1, Figure S1: Predicted secondary structure of the *rrnL* in the mitogenome of *C. fenestrata*. Figure S2: Predicted secondary structure of the *rrnS* in the mitogenome of *C. fenestrata*. Figure S3: Heuristically exploring the mitochondrial rearrangements of *C. fenestrata* and *E. polynesia*. Figure S4: Summary of the major clades recovered by different datasets and analytical approaches. Figure S5: Phylogenetic relationship inference using different datasets and analytical approaches. Table S1: Summary of mitogenome data used in this study. Table S2: Comparison of the annotated mitochondrial genomes of *C. fenestrata* and *E. polynesia*. Table S3: Nucleotide composition of the mitogenome of *C. fenestrata* and *E. polynesia*. Table S4: The relative synonymous codon usage (RSCU) of PCGs in Anthophila mitogenomes. Table S5: Microsatellite-like identification in the putative control region of the mitogenome of *C. fenestrata* and *E. polynesia*. Table S6: Pairwise common intervals comparison of mitochondrial gene orders among Anthophila species, based on the order of all tested genes. Table S7: Sliding window analyses of the mitogenome of *C. fenestrata*, *M. sculpturalis*, *E. Polynesia* and *M. strupigera*. Table S8: Saturation substitution tests for PCGs, rRNAs and tRNAs of the mitogenomes of Anthophila.
