# Supplementary material for: Comparative Mitogenomic Analysis of Two Cuckoo Bees (Apoidea: Anthophila: Megachilidae) with Phylogenetic Implications
Source: Insects. 2021 Jan 5;12(1):29. doi: 10.3390/insects12010029 (PMC7824771; doi:10.3390/insects12010029)
Supplement: Supplementary file 1 [file insects-12-00029-s001.zip › Supplementary Files/Figure S2 Predicted secondary structure of the rrnS in the mitogenome of C. fenestrata.pdf]

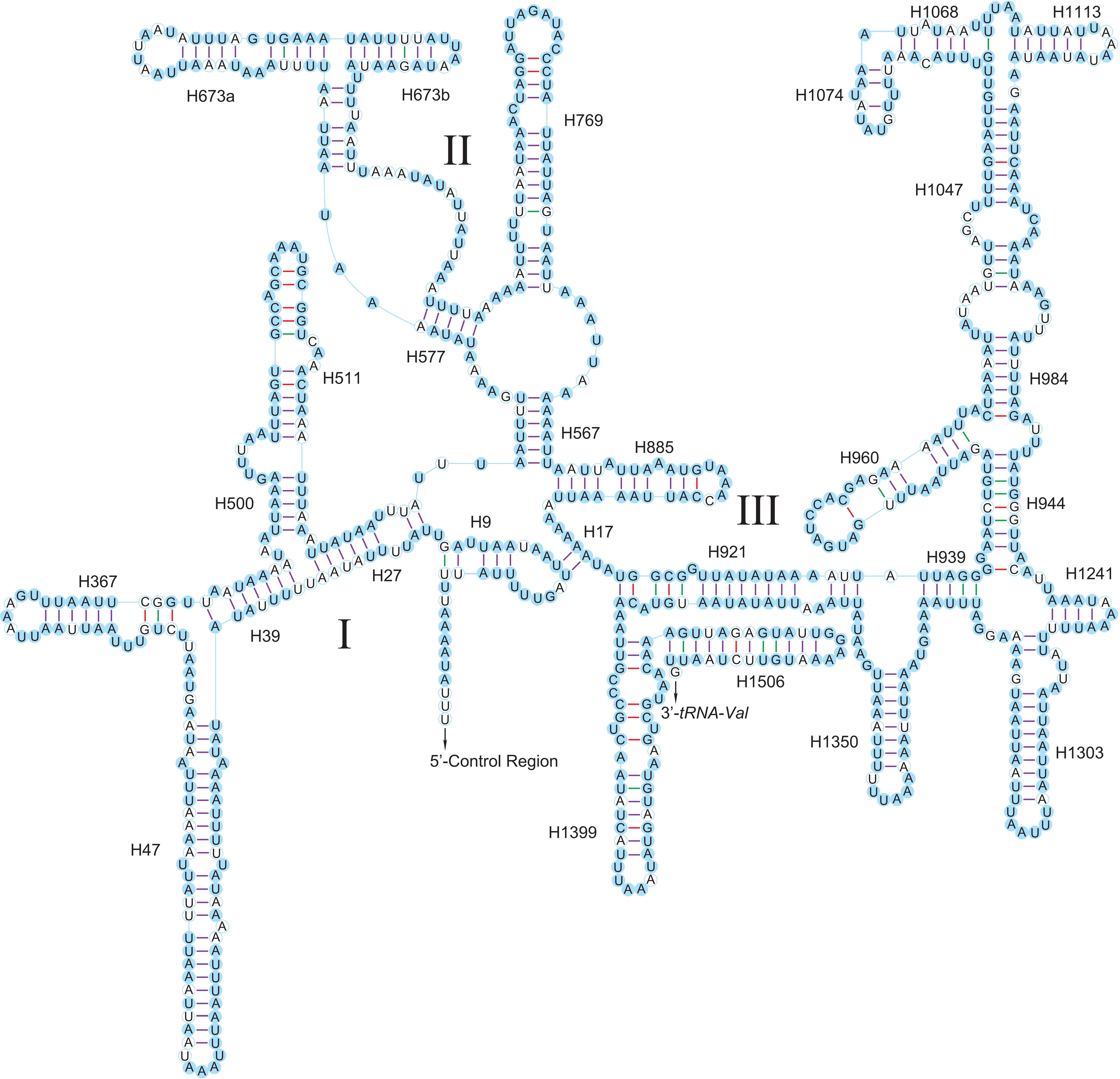

Note Predicted secondary structure of the *rrnS* in the mitogenome of *C. fenestrata. Filled circle, nucleotide conserved in in E. polynesia mitogenomes.* hollowed circle, nucleotide not conserved. A-U bonds, G-U bonds, G-C bonds and mismatches are represented by the purple line, the green line, the red line and the solid dots, respectively.
