# Supplementary material for: Comparative Mitogenomic Analysis of Two Cuckoo Bees (Apoidea: Anthophila: Megachilidae) with Phylogenetic Implications
Source: Insects. 2021 Jan 5;12(1):29. doi: 10.3390/insects12010029 (PMC7824771; doi:10.3390/insects12010029)
Supplement: Supplementary file 1 [file insects-12-00029-s001.zip › Supplementary Files/Figure S3 Heuristically exploring mitochondrial rearrangements of C. fenestrata and E. polynesia.pdf]

CREx: comparison

back to distance matrix

Ancestral type → Euaspis polynesia

- family diagram for Ancestral type (e)

  CR I -Q M nad2 W -C -Y cox1 L2 cox2 K D atp8 atp6 cox3 G nad3 A R N S1 E -F -nad5 -H -nad4 -nad4L T -P nad6 CytB S2 -nad1 -L1 -rrnL -V -rrnS

- family diagram for Euaspis polynesia (e)

  CR A I -Q nad2 -Y W cox1 L2 cox2 D K atp8 atp6 cox3 G nad3 -C -M -R N S1 E -F -nad5 -H -nad4 -nad4L T -P nad6 CytB S2 -nad1 -L1 -rrnL -rrnS -V

- scenario:
  - transposition

K D	(e)
D K	(e)

  - transposition

I -Q M nad2 W -C -Y cox1 L2 cox2 D K atp8 atp6 cox3 G nad3 A	(e)
A I -Q M nad2 W -C -Y cox1 L2 cox2 D K atp8 atp6 cox3 G nad3	(e)

  - transposition

-V -rrnS	(e)
-rrnS -V	(e)

  - reverse transposition

M nad2 W -C -Y cox1 L2 cox2 D K atp8 atp6 cox3 G nad3	(e)
nad2 W -C -Y cox1 L2 cox2 D K atp8 atp6 cox3 G nad3 -M	(e)

  - reversal

R	(e)
-R	(e)

  -

transposition	W -C -Y cox1 L2 cox2 D K atp8 atp6 cox3 G nad3	(e)
→	-C -Y W cox1 L2 cox2 D K atp8 atp6 cox3 G nad3	(e)
transposition	-C -Y W cox1 L2 cox2 D K atp8 atp6 cox3 G nad3	(e)
→	-Y W cox1 L2 cox2 D K atp8 atp6 cox3 G nad3 -C	(e)

Ancestral type → Coelioxys fenestrata

- family diagram for Ancestral type (e)

  CR I -Q M nad2 W -C -Y cox1 L2 cox2 K D atp8 atp6 cox3 G nad3 A R N S1 E -F -nad5 -H -nad4 -nad4L T -P nad6 CytB S2 -nad1 -L1 -rrnL -V -rrnS

- family diagram for Coelioxys fenestrata (e)

  CR -C A M I -Q nad2 -Y W cox1 L2 cox2 D K atp8 atp6 cox3 -R G nad3 N S1 E -F -nad5 -H -nad4 -nad4L T -P nad6 CytB S2 -nad1 -L1 -rrnL -V -rrnS

- scenario:
  - transposition

I -Q M	(e)
M I -Q	(e)

  - transposition

K D	(e)
D K	(e)

  -

reversal	M I -Q nad2 W -C -Y cox1 L2 cox2 D K atp8 atp6 cox3 G nad3 A R	(e)
→	M I -Q nad2 W -C -Y cox1 L2 cox2 D K atp8 atp6 cox3 G nad3 A -R	(e)
tdrl	M I -Q nad2 W -C -Y cox1 L2 cox2 D K atp8 atp6 cox3 G nad3 A -R	(e)
→	W -C cox1 L2 cox2 D K atp8 atp6 cox3 A -R M I -Q nad2 -Y G nad3	(e)
tdrl	W -C cox1 L2 cox2 D K atp8 atp6 cox3 A -R M I -Q nad2 -Y G nad3	(e)
→	-C A M I -Q nad2 -Y W cox1 L2 cox2 D K atp8 atp6 cox3 -R G nad3	(e)
