# Supplementary figures and images for: Comparative Mitogenomic Analysis of Two Cuckoo Bees (Apoidea: Anthophila: Megachilidae) with Phylogenetic Implications
Source: Insects. 2021 Jan 5;12(1):29. doi: 10.3390/insects12010029 (PMC7824771; doi:10.3390/insects12010029)

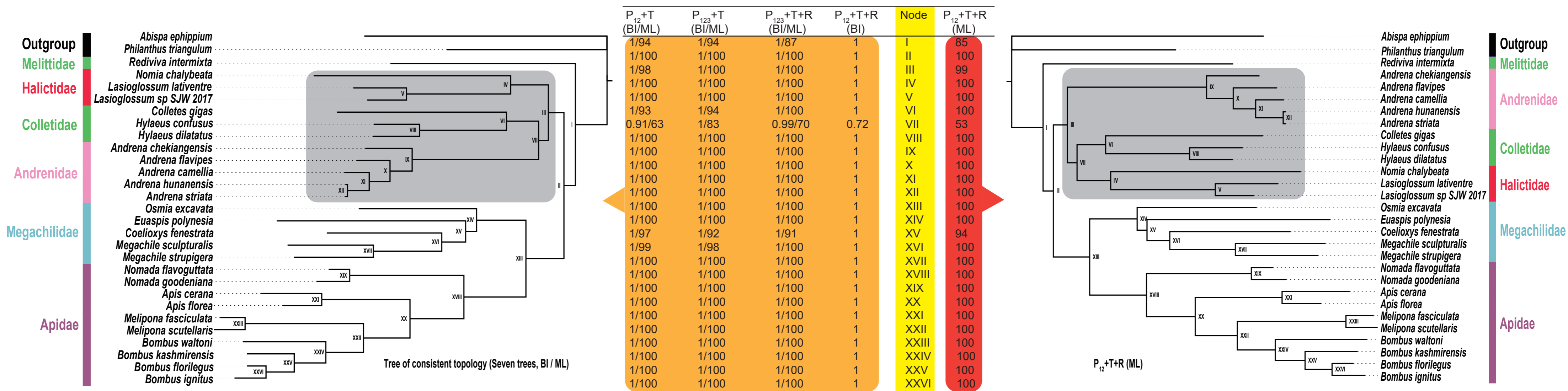

Supplement: Supplementary file 1 [file insects-12-00029-s001.zip › Supplementary Files/Figure S4 Summary of the major clades recovered by different datasets and analytical approaches.pdf]

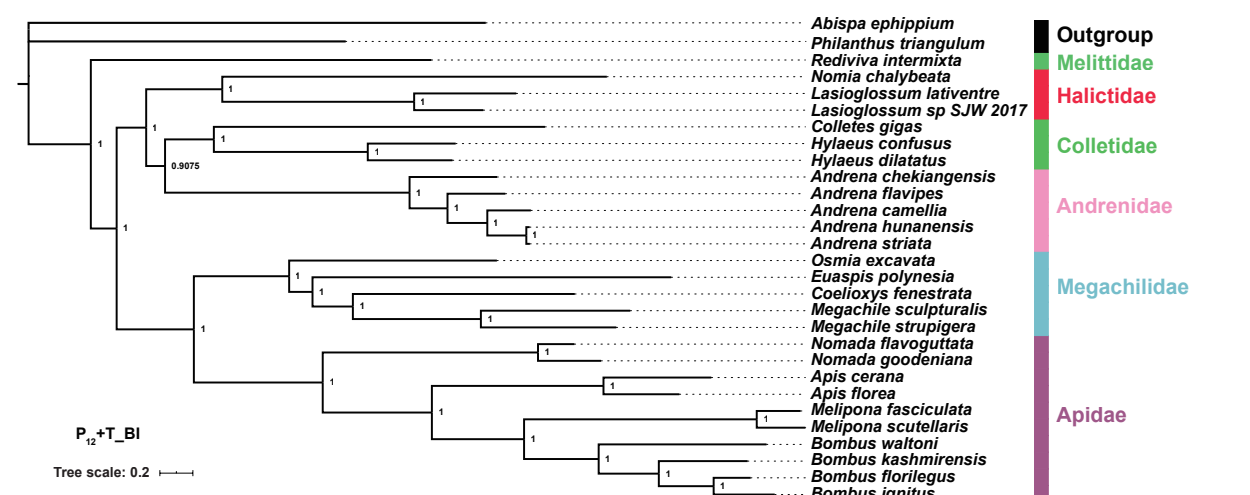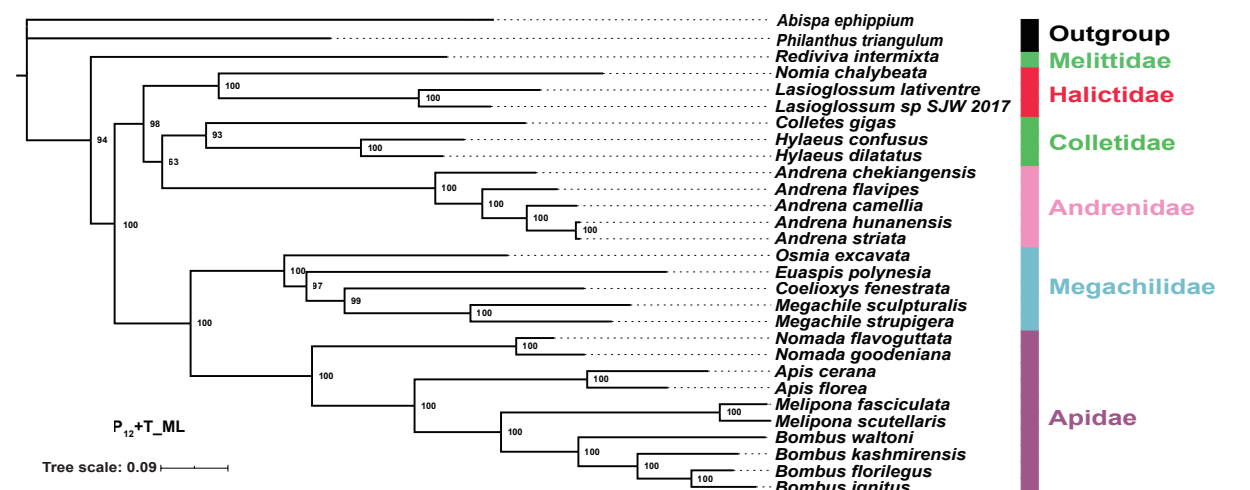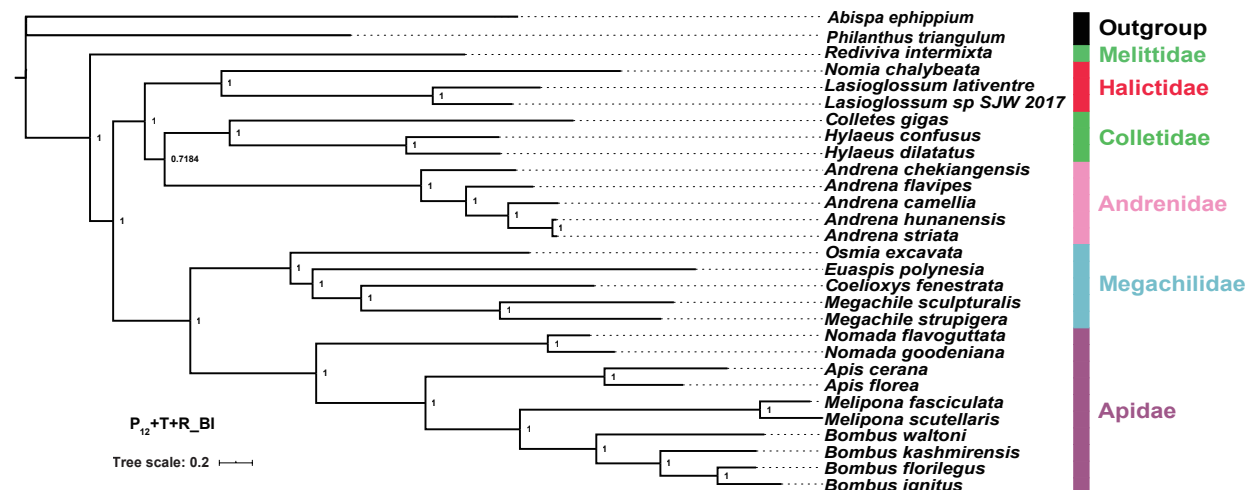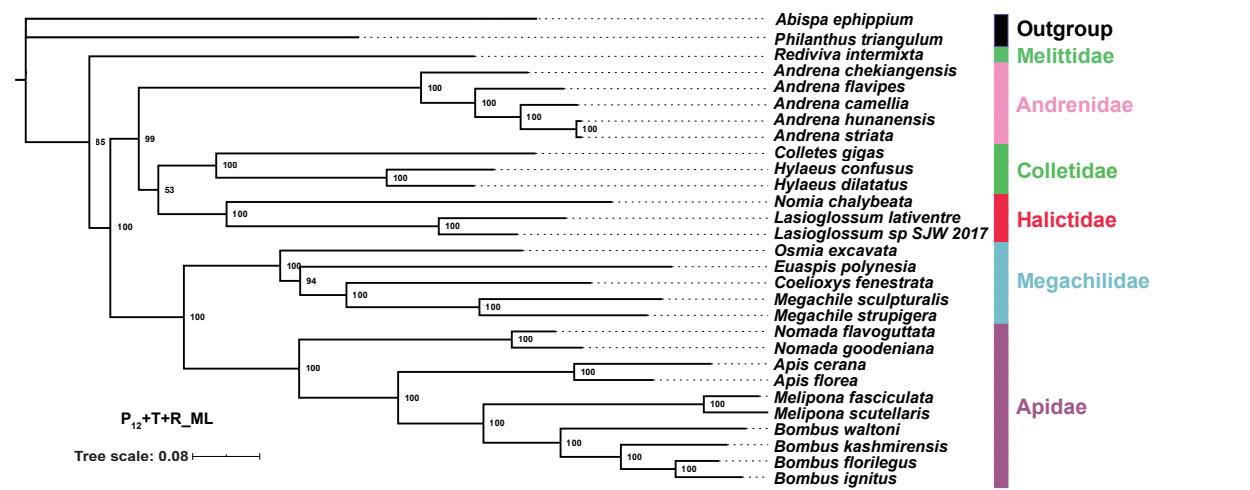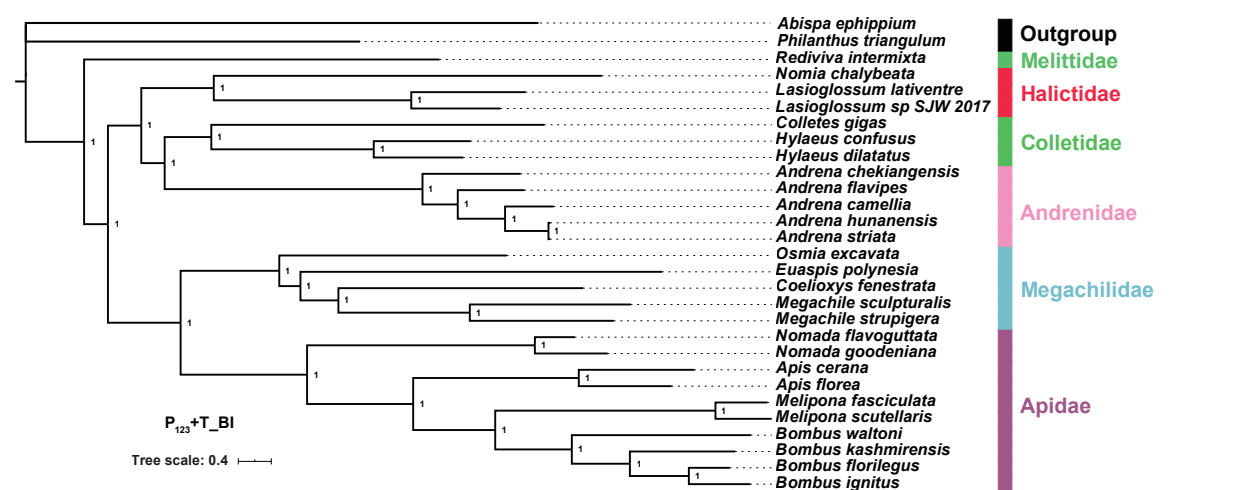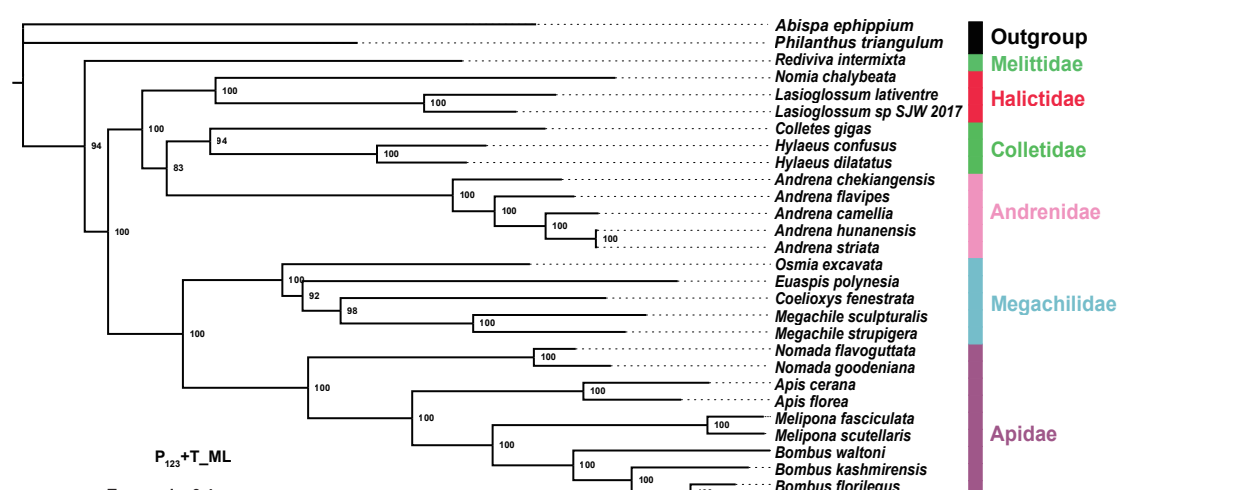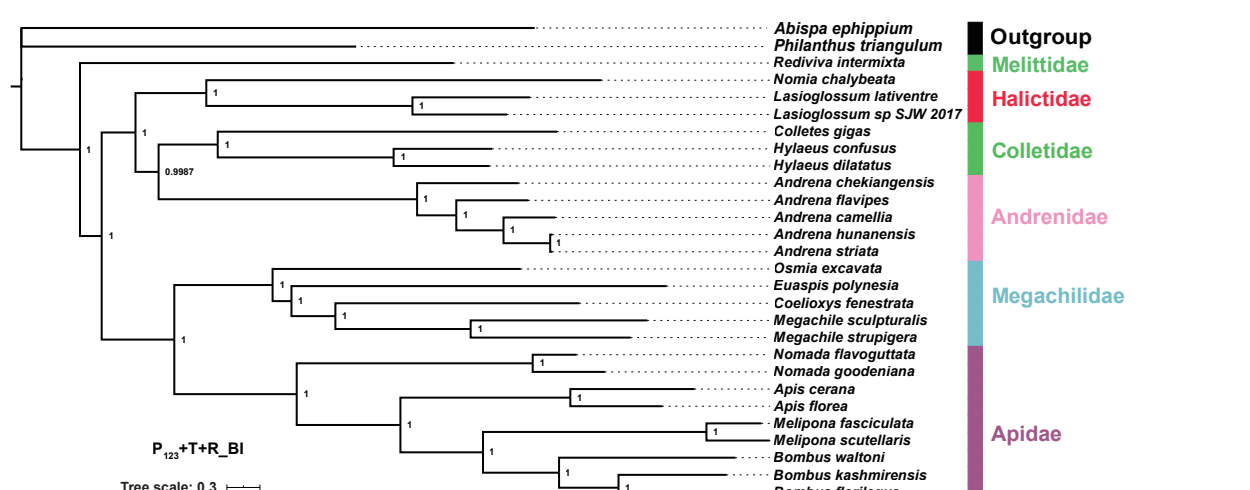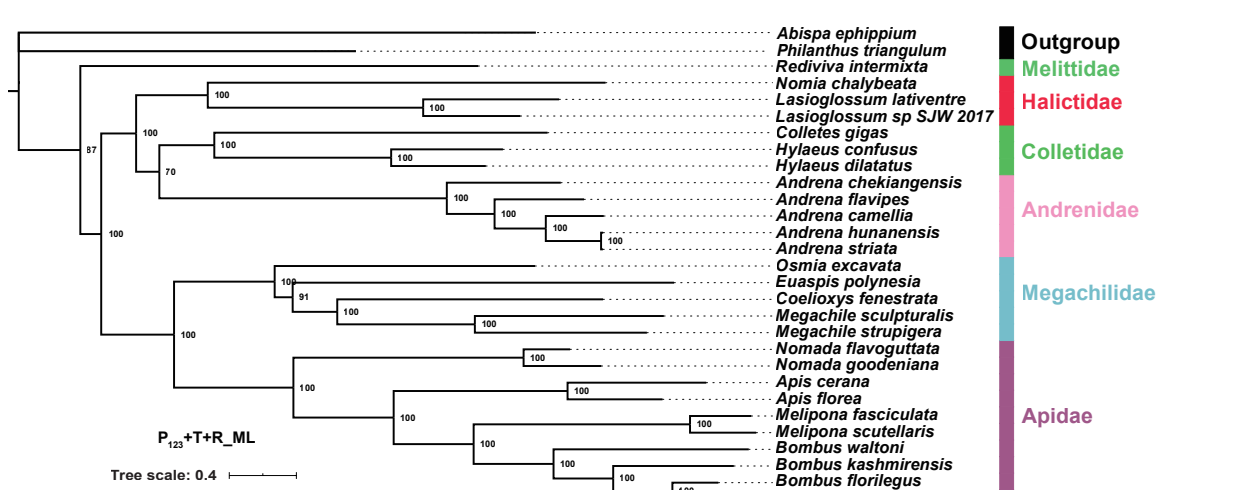

Supplement: Supplementary file 1 [file insects-12-00029-s001.zip › Supplementary Files/Figure S5 Phylogenetic relationship inference by different datasets and analytical approaches.pdf]
