# Supplementary material for: Comparative Mitogenomic Analysis of Two Cuckoo Bees (Apoidea: Anthophila: Megachilidae) with Phylogenetic Implications
Source: Insects. 2021 Jan 5;12(1):29. doi: 10.3390/insects12010029 (PMC7824771; doi:10.3390/insects12010029)
Supplement: Supplementary file 1 [file Figure S1 Predicted secondary structure of the rrnL in the mitogenome of C. fenestrata.pdf › Supplementary Files/Figure S1 Predicted secondary structure of the rrnL in the mitogenome of C. fenestrata.pdf]

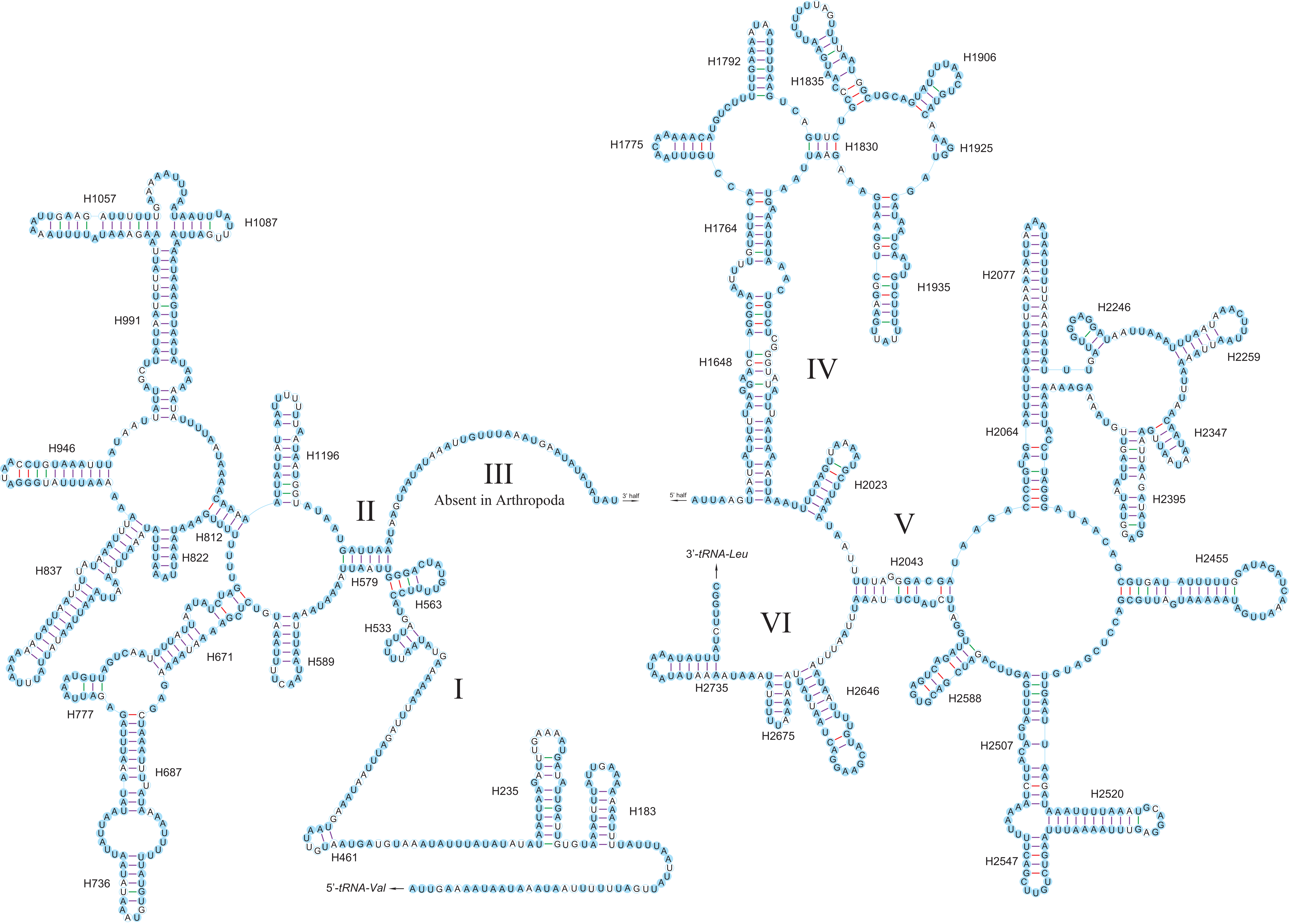

Note Predicted secondary structure of the *rrnL* in the mitogenome of *C. fenestrata. Filled circle, nucleotide conserved in in E. polynesia mitogenomes.*; hollowed circle, nucleotide not conserved. A-U bonds, G-U bonds, G-C bonds and mismatches are represented by the purple line, the green line, the red line and the solid dots, respectively.
